# Validating Wound Severity Assessment via Region-Anchored Convolutional Neural Network Model for Mobile Image-Based Size and Tissue Classification

**DOI:** 10.3390/diagnostics13182866

**Published:** 2023-09-06

**Authors:** Yogapriya Jaganathan, Sumaya Sanober, Sultan Mesfer A Aldossary, Huda Aldosari

**Affiliations:** 1 Department of Computer Science and Engineering, Kongunadu College of Engineering and Technology, Trichy 621215, India; 2 Department of Computer Science, Prince Sattam Bin Abdulaziz University, Wadi al dwassir 1190, Saudi Arabia; hu.aldosari@psau.edu.sa; 3 Department of Computer Sciences, College of Arts and Sciences, Prince Sattam Bin Abdulaziz University, Wadi al dwassir 1190, Saudi Arabia; s.aldossary@psau.edu.sa

**Keywords:** wound care, deep learning, image validation, superpixel, mobile device, decision-making and region of interest

## Abstract

Evaluating and tracking the size of a wound is a crucial step in wound assessment. The measurement of various indicators on wounds over time plays a vital role in treating and managing crucial wounds. This article introduces the concept of utilizing mobile device-captured photographs to address this challenge. The research explores the application of digital technologies in the treatment of chronic wounds, offering tools to assist healthcare professionals in enhancing patient care and decision-making. Additionally, it investigates the use of deep learning (DL) algorithms along with the use of computer vision techniques to enhance the validation results of wounds. The proposed method involves tissue classification as well as visual recognition system. The wound’s region of interest (RoI) is determined using superpixel techniques, enabling the calculation of its wounded zone. A classification model based on the Region Anchored CNN framework is employed to detect and differentiate wounds and classify their tissues. The outcome demonstrates that the suggested method of DL, with visual methodologies to detect the shape of a wound and measure its size, achieves exceptional results. By utilizing Resnet50, an accuracy of 0.85 percent is obtained, while the Tissue Classification CNN exhibits a Median Deviation Error of 2.91 and a precision range of 0.96%. These outcomes highlight the effectiveness of the methodology in real-world scenarios and its potential to enhance therapeutic treatments for patients with chronic wounds.

## 1. Introduction

Skin ulcers, a leading source of disease and mortality around the globe, are brought on by a number of illnesses and disorders, including insulin resistance, peripheral nerve damage, motionlessness, stress, artery disease, illnesses, and vein insufficiency. These ulcers are characterised by their inability to heal properly within the expected timeframe of 4 weeks to 3 months, leading to a lack of functional and anatomical restoration. This is particularly relevant in the case of diabetic foot ulcers. Underlying pathological factors often hinder or delay the healing process, resulting in significant negative impacts on the physical, emotional, and social well-being of affected individuals. Chronic ulceration and foot diabetes amputated ulcer demand a lot of time and supplies for treatment, which has economic ramifications [1]. These variables contribute to the worldwide difficulty in treating complicated wounds like pressure ulcers, lesions of the lower extremities (such as arterial, venous, and diabetic foot ulcers), and neoplasm lesions [2]. Due to the absence of standardised treatment recommendations and the abundance of care items, it is possible that the root cause of complicated injuries is not adequately addressed during therapy. The engagement of healthcare experts is critical for the medical care of complicated injuries in order to encourage regeneration and enhance the use of both physical and mental assets. Clinical practise rules for managing wounds have been modified by medical organisations by adding additional assistance systems. These systems act as tools to aid healthcare providers in their organisational and behavioural decisions [3]. These recommendations’ main goal is to provide doctors with the information they need to identify, avoid, and manage injuries as effectively as possible. In order to improve the efficiency of the recovery process, they also advocate for the careful allocation of organisational capabilities. Medical practise recommendations often concentrate on resolving problems associated with the avoidance and therapy of wounds caused by pressure in those that are vulnerable or have been diagnosed with them presently. These guidelines outline the various stages of clinical practice involved in managing chronic wounds, encompassing prevention, treatment, and healing [4].

Chronic wounds contribute significantly to illness and death rates, placing a substantial financial burden of around $25 billion per year on the U.S. healthcare system. Effective management of patients with chronic wounds involves regular visits by an interprofessional team. The primary measure of wound healing is the reduction in the surface area of the lesion. This measurement is essential for clinicians to track progress and make informed decisions regarding treatment options. Therefore, precise and accurate wound area measurements play a vital role in maximizing patient outcomes for individuals with chronic wounds. Many medical facilities still use mechanical ruler-based measures [5,6], which are inconsistent and might underestimate the wound’s extent by 40%. Reach acetic tracer is a different frequently utilised technique, although it may change the boundaries of the wound, put individuals at a greater chance for disease at danger, and be uncomfortable. Automated analogue planimetry, which analyses wound pictures, increases precision but is time-consuming and may not be suitable for busier healing facilities. The proportion of normal granulation tissue in the site of the wound, in addition to the overall size of the lesion, is a key factor in deciding when a wound will start to recover or if it has to be definitively closed with a skin transplant or flap [7,8,9]. Clinicians use colour to determine the proportion of tissue that granulates (PGT). Dark red tissue with granules may signify disease, whereas pale tissues could indicate inadequate neurogenesis and blood flow to the wound bed. Visual assessment of PGT is, however, unreliable and might vary greatly amongst doctors. According to current studies, colour picture analysis of granulation tissues can forecast how the ulcers will heal, offering a more precise evaluation than perceptual judgement alone.

This has yet to be a widely accepted methodology for assessing injuries. However, developments in AI are making automatic diagnostics evaluation of images possible. As wound diagnostic tools and instruments like Silhouette (Aranz Medicine) and insight (Ekare) get better, doctors are able to evaluate injuries more quickly, accurately, and consistently. This helps doctors make better decisions and gives patients better results. However, rigorous validation is necessary to ensure that these tools perform comparably to standard measurement techniques. AI-based visual injury detection solutions are not clinically evaluated.

This study seeks to evaluate the dependability of AI-driven injury assessment technology by juxtaposing it against the judgments made by medical professionals through manual evaluations. The effectiveness of the AI in accurately identifying wound areas featuring granulation tissue was measured by thoroughly analysing the error metrics pattern between the AI-generated tracings and a benchmark established by two individual human tracings. However, we acknowledge that relying solely on two human evaluators might have limitations, and we recognize the importance of considering alternative, clinically validated strategies for a more comprehensive validation process. We also created a qualitative technique for assessing the AIs efficiency since wound evaluation is biased. This featured skilled wound healing, presenting doctors masked-reviewing AI and humans tracings.

### 1.1. Problem Statement

The problem addressed in the research paper titled “Wound Image Validation using Region Anchored CNN Model for tracking size and tissue classification” revolves around the accurate and reliable validation of wound images, specifically concerning two critical aspects: wound size tracking and tissue classification. In order to deal with injuries successfully and to plan treatments, it is crucial to identify the various kinds of tissue present in an injury and to assess them precisely. This study intends to address the issues associated with injury imagery analysis.

Existing wound image validation methods may suffer from inconsistencies, subjective assessments, and manual errors, making it difficult to obtain accurate and standardised measurements. Manual tracing and visual estimation can lead to variations between clinicians, impacting the reliability of wound assessments and decisions regarding treatment options. Therefore, there is a need for an advanced and automated approach that can consistently and objectively track wound size and classify different tissue types in wound images.

### 1.2. Major Contribution of the Proposed Research Work

The effective adoption of the Region Anchored CNNs Algorithm for injuries’ picture evaluation might impact wound treatment and care. Major contributions of the above research work can be summarised as follows:

- Leveraging mobile device-captured photographs, our approach offers a convenient and accessible way to monitor wound size changes over time.
- By integrating deep learning algorithms and computer vision techniques, our method aims to improve wound validation outcomes, aiding healthcare professionals in making well-informed decisions for patient care.
- The proposed approach employs a Region Anchored Convolutional Neural Network (CNN) framework, utilizing superpixel techniques to accurately identify the wound’s region of interest (RoI), leading to precise wound size calculations.
- Beyond wound size, our research incorporates a Tissue Classification CNN that effectively categorises different tissue types within the wound.
- The model’s Median Deviation Error of 2.91 and precision range of 0.96% highlight its success in accurately detecting and classifying diverse tissue types, including granulation tissue, necrotic tissue, and epithelial tissue.

## 2. Review of Related Works

Wounds can be classified in various ways, including by wound type, wound tissue, burn extent, and more. When categorising wound types, both wounds and non-wound areas (such as normal skin or background) are considered. Binary wound type classification examples include distinguishing between background and diabetic foot ulcers (DFU), normal epidermis and pressure ulcers (PU), or DFU and PU. On the other hand, wound type classification with multiple classes includes differentiating among DFU, PU, and venous leg ulcers (VLU). An injury tissues categorisation requires recognising granulation, slough, and cremation. Burn thickness category classifies burn lesions as superficial in nature, deep, or full-thickness. This section examines the data-driven approaches currently in use to identify injury patterns as the present research is primarily concerned with that topic. ML and DL are used to classify injury patterns in these investigations.

A ML method for differentiating burn injuries from ulcers created by pressure is developed [10]. The researchers used DL structures that have already been trained, such as VGG-face, ResNet101, and ResNet152, to identify characteristics from the photos. After that, a SVMs classification was used to divide the photos into groups for wounds from pressure and burns. This research utilised burn along with stress injury photographs from hospitals and online sources. Three categories—burn, tension, and healthy epidermis—were added to the collection, totaling 990 photos. There were other tests carried out, which included a binary distinction (differentiating among stress and burnt) and three-class categorisation (differentiating among tension, burn, and good epidermal).

Researchers conducted a study using conventional MLs, DLs, and CNNs models to perform binary classification on images of diabetic foot ulcers (DFUs) [11]. The classification tasks included distinguishing between ischemic and non-ischemic ulcers, as well as identifying infected and non-infected ulcers. 

The Bayes Net, RF, and MP procedures were used by the researchers for traditional ML. Three CNN networks—InceptionV3, ResNet50, and InceptionResNetV2—were used in DL techniques. The three CNNs systems’ bottlenecks characteristics were retrieved and used as inputs by the SVM-based classifier that made up the combined CNNs. The collective CNN outperformed both DL systems and classical artificial intelligence approaches throughout the process of assessment, with its best accuracy in both categorical categories. The researchers reported an accuracy rate of 90% for ischemic detection and a 73% efficiency rate for pathogen detection.

A stacking ensemble model based on multi-mRNA data is focused on wound age estimation [12]. The authors used methods of data enhancement to increase the dataset size and used a dataset of 397 photos of wounds. The results from three parallel convolutional networks with various filter widths were combined using the ensemble framework. The suggested method’s outstanding reliability at a rate of 92.5% was attained.

An overview of clinical examinations for evaluating arterial and venous insufficiency was presented using a CNN-based method [13]. The research made use of a pre-trained VGG-19 system that was developed particularly to divide ulcer pictures into the two aforementioned groups. A collection of 300 photos that had been labelled by an injury expert was used prior to the network activation. Methods for data augmentation and preparation have been employed to improve the raw data. The VGG-19 system was subsequently tuned utilising dermoscopic pictures. For the categorisation test comparing VLUs with non-VLUs, the authors achieved a success rate of 85%, accuracy of 82%, with recollection of 75%.

A CNN-based classification approach for lesion images [14]. They compiled a collection of 1335 injury images using online and cellphone resources. The database had nine designations, each one containing two separate subcategories (positively and negatively). Wound, infections (SSI), tissue with granulation, fibrinous fluids, exposing injury, water drainage, steri segments, essentials, and closures were among the labels. WoundNet, an improved VGG16 system learned on ImageNet, was used as the classification. Furthermore, they created a team approach known as Deepwound that aggregated the outcomes of three separate models. The “wound” category had a 82% reliability, compared to 72% for drainage and 97% for steri strip. On the other hand, Guerra and coworkers [13] suggested a CNN-based approach for separating pictures of ulcers from non-ulcer sources including VLUs. They used a pre-trained VGG-19 system programmed to categorise ulcer photos into both of the aforementioned groups. An injury expert evaluated 300 photos for the dataset, which received data preparation and supplementation. The VGG-19 system was then fine-tuned utilising dermoscopic pictures. The researchers achieved 85% reliability, 82% preciseness, and 75% recollection for the VLU vs. non-VLU categorisation challenge.

In order to conduct binary patch distinctions between healthy skin against aberrant skin, especially ulcers from DFU, Ref. [15] built a unique DCNNs named DFU_QUTNet. They originally presented a novel database made up of 754 foot pictures that were acquired from an Iraqi institution that specialised in treating diabetes patients. Within this dataset, they generated 542 patches representing normal skin and 1067 patches representing DFU. To augment the training samples, they applied transformations such as flipping, rotating, and scaling, resulting in a multiplication of the training samples by a factor of 13. Their approach was compared with GoogLeNet, VGG16, and AlexNet to assess its effectiveness. They were able to reach an optimal F1-Score of 94.5 percent using the SVMs in conjunction with the DFU_QUTNet framework.

Using a DCNNs based classification algorithm, Ref. [16] suggested categorising complete lesion pictures into a variety of groupings, such as surgical, suffering from diabetes, and ulcers of the veins. In their technique, the resultant ratings of two classifiers—one determined by patch-wise evaluation and one that relied on image-wise analysis—were combined. The Multi-Layer Perceptron (MLP), which was used to create a more trustworthy and precise classification algorithm, was then given these results. The study introduced a novel dataset comprising 538 authentic wound images, representing four distinct wound types. For binary classification tasks, the maximum and average accuracy values were reported as 96.8% and 95.29%, respectively. For the three-class classification task, the maximum and average accuracy values were reported as 91.9% and 87.7%, respectively. 

Researchers [17] classified persistent injuries into four distinct categories, diabetic, lymphovascular, injury from pressure, and surgical, using an understandable AIs technique. In total, 8690 disease photos from eKare, Inc.’s repository of data made up their dataset. They used methods including mirroring, movement, and vertical reversal to enhance the quantity of injured pictures and equalise their distribution among categories. They used transfer learning to train on the VGG16 net for their classifier. The researchers observed a mean F1 test result of 0.76. The XIA technique made the wound image classification comprehensible and transparent, making it possible to comprehend the reasoning behind the algorithm’s classifying choices for a given class. Additionally, although earlier research has attempted to categorise different kinds of injuries using injury photos, as far as we are aware, no studies have attempted to categorise injuries depending just on where they are. This research is the first study to use the exact position of the injury as a consideration in the automated categorisation of wounds. It presents a multimodal network that uses wound pictures and location-related parameters to categorise wounds effectively. The overview of the chapter on associated research is shown in Table 1.

### Research Gaps Identified

Based on the above survey on wound classification using machine learning and deep learning techniques, the following research gaps are identified:

- Many preceding studies have concentrated on specific wound types and datasets derived from particular healthcare institutions. This narrow focus hampers the broader applicability of their developed models. It is crucial to bridge this gap by creating models that encompass a wider range of wound types and datasets, enabling their deployment across diverse clinical scenarios.
- Existing approaches often centre around offline wound classification, relying on static images for analysis. A critical gap emerges in the absence of real-time wound assessment solutions capable of tracking dynamic changes in wound appearance and monitoring healing progress over time. Addressing this gap involves developing methods that adapt to evolving wound conditions and provide real-time insights.
- Several studies demonstrate promising outcomes in terms of wound classification accuracy. However, a notable gap remains in determining the feasibility of implementing these models in real-world clinical settings. This involves considering hardware limitations, privacy concerns, and integration challenges with existing healthcare systems. Research efforts are required to ensure that effective models can be smoothly incorporated into the healthcare workflow.

By addressing these research gaps, the proposed model will contribute to advancing the field of wound classification by using Region Anchored CNN framework which leads to more effective and efficient wound management and improved patient outcomes with their smartphone captured wound images.

## 3. Taxonomy of the Proposed Methodology

**Figure 1:**
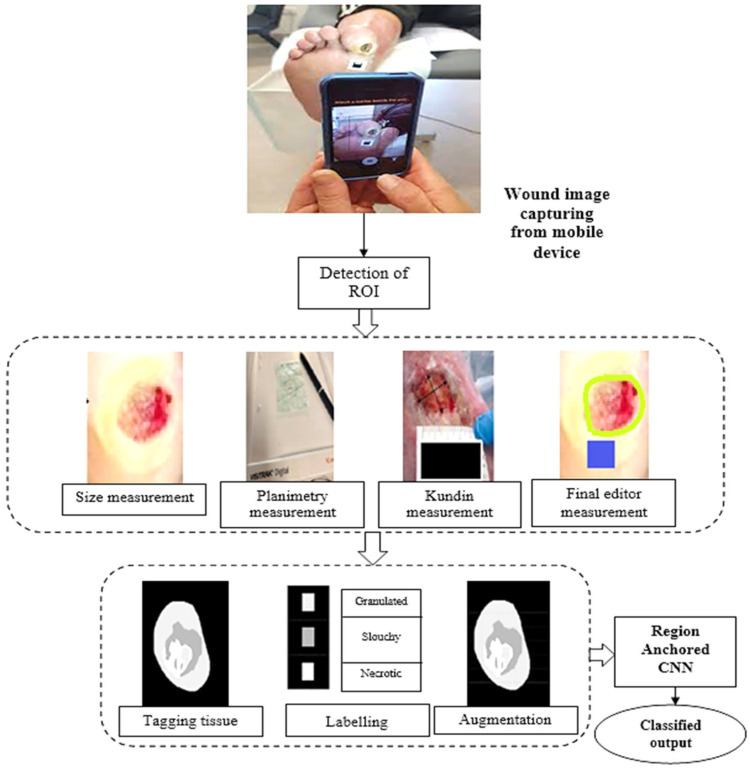
Pipeline of the proposed model flow.

This research introduces a digital approach for determining the area of a lesion and categorising its tissues. A computerised device selects the location that is important (defining the wound), a computerised method measures space utilising a third-party calibrator, and an experienced convolutional neural structure classifies tissue. Figure 1 shows the information gathering and sorting stages, which includes evaluation and tissues grouping. These processes were integrated into clinical practice using the clinic gram^®^ mobile application.

### 3.1. Wound Identification Mechanism

**Figure 2:**
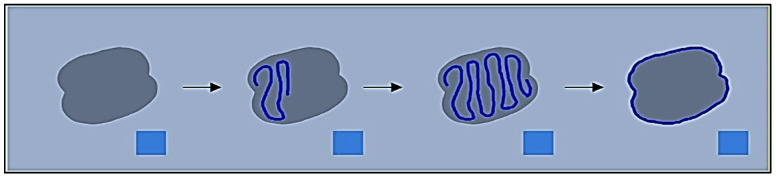
Capturing, highlighting, and extracting the wound mask from a test image allows the system to detect a wound.

**Figure 3:**
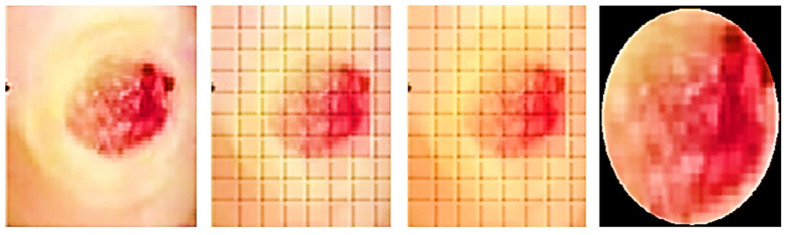
Original, superpixel segmented, scraw, and identified region of interest images.

This method uses neighbouring pixels’ similarity to determine a wound’s boundaries. When recording a picture of an injury utilising a smartphone or tablet, the individual creates a scribble inside the injury zone (refer to Figure 2) to assist the technology in recognising the injury. The procedure is shown using an example picture in Figure 3. After the machine locates the shape of the injury, a surgical mask [18] is used to separate the area that is important from the backdrop and epidermal around it.

In our proposed approach, the identification of the Region of Interest (ROI) within wound images is a pivotal step for accurate wound assessment. The ROI corresponds to the area of the image that encompasses the wound itself, excluding surrounding healthy tissue. This process involves the utilization of a Region Anchored Convolutional Neural Network (CNN) framework that employs superpixel techniques to pinpoint the exact boundaries of the wound within the image.

Superpixel Segmentation: Superpixel segmentation is a technique that groups pixels with similar characteristics, such as colour and texture, into coherent regions. This approach assists in effectively identifying the boundaries of the wound, as it considers both local and global image features. By segmenting the image into superpixels, the CNN can then focus its attention on these localised regions, aiding in the precise delineation of the wound area.

Region Anchoring: The Region Anchored CNN model leverages the information from superpixels to anchor and align the CNN’s receptive field with the wound’s boundaries. This anchoring process ensures the CNN’s analysis to be concentrated on the wound area, which subsequently enables accurate identification of the ROI. By integrating superpixel segmentation and region anchoring within the CNN framework, our approach ensures that the wound’s ROI is precisely identified. This is paramount for accurate wound size calculations, tissue classification, and overall effective wound assessment.

The approach uses a mix of the super pixel approach and k-means algorithm [19] to figure out the shape of the injury and describe the ROIs after trying out approaches like Felzenszwalb [4], Mean shift, as well as Rapid change [6]. The super pixel technique dynamically divides the wound picture into many segments that have comparable characteristics or traits. In contrast, the unsupervised categorisation (clustering) method k-means organises objects according to their features. By minimising the sum of similarities among each item to the centre of its cluster, grouping is accomplished. Quadratic proximity is often used in this process. The system applies those algorithms and splits the picture into several megapixels to determine the shape of the injury. FNRs comprised the reference tracing region included located in the specimen’s testing observe established by referencing traced overall region utilising calculation 1.

(1) FNR=100×R−O/R

The healthcare expert sketches a tiny portion within the injury utilising the identical cellphone programme that was employed for picture collection, and the outline is created by relating several megapixels of the injury. The SP algorithm operates automatically and autonomously to calculate the various superpixelblocks. This section highlights the importance of adjusting the different operating variables of SP for optimal performance. These elements consist of N sections: The approximate number of sections needed. When grouping images in SP, compact influences how colour and closeness are compared. Greatest repetitions are permitted by the k-means algorithm. The Gaussian picture blurring filter’s symbol is sigma. Enforce connectivity: A variable indicating whether or not the segments should be connected. The adjustment of these variables is crucial to achieving the best results in detecting the wound’s contour.

### 3.2. Wound Area Calculation

**Figure 4:**
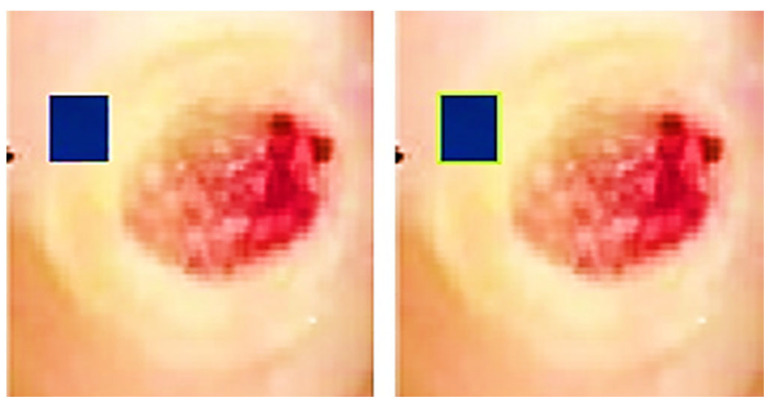
Left: original wound and marker, right: marker identified with green outline.

**Figure 5:**
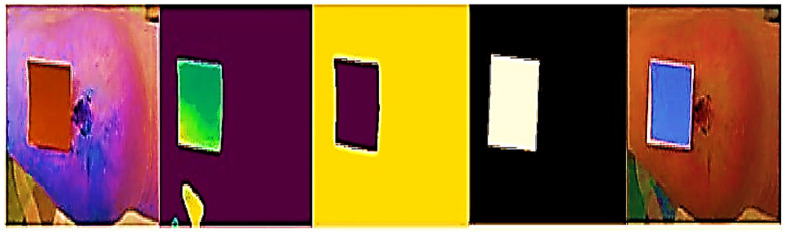
Left to right: marker detection and corrected picture processing.

The ROIs and an indicator are used to measure the incision. A blue rectangle (see Figure 4) serves as our identifier because of its high contrast with the background. Each side of the square marker is 2 cm long, giving it a set size. We can figure out how big the cut is by compared the sizes of the line on the picture to how big it is in real life. The doctor places the marker perpendicularly close to the incision before taking the picture. This step is essential since the marker’s precise placement affects the measured results. The measurement is deemed to be inaccurate if the indicator continues to be inclined, partly concealed, or impacted by light or shadow. In the first step (see Figure 5), the picture is read and converted from RGBs to HSVs colour space. The picture is then restricted to a range of colours, including many shades of blue, producing a mask which just contains pixels inside that spectrum. The mask is next checked for object features, and only squares with lengths greater than twice their breadth, like our indicator, are picked. An artificial eye algorithm is then used to roughly convert all of the outlines into square forms. The function determines which chosen rectangular forms most closely resemble the real contour through assessing the regions’ sizes. If numerous square forms are discovered, the form that is nearest to a rectangular shape, indicating the ones with the smallest distinction among the object’s region after performing the function and the originally identified region with the mask being used, is kept.

Furthermore, an evaluation system for the marker has been implemented to ensure accurate measurements. The technology assesses the marker’s proportionality and asymmetricity to find its right place. The measurement of assessment is created by dividing the lesser identified half by the bigger identified sides in order to measure disparity. A score close to 0 denotes extreme disproportion, whereas an amount close to 1 represents a perfect structure with optimal proportions. Calculating vertical as well as horizontal disparity requires the marker’s vertex’ tilt. If the marker [20] is positioned entirely along the X and Y axes, this measurement tends towards 0. If the Z-axis is horizontal, the imbalanced values on both sides ought to be identical. The dimensions of the indicated incision may be determined by calculating the ratio received from the indicator, which connects the total amount of pixels to the real measurement of 4 cm^2^, after the indicator has been identified. After being found, the precision of the percentage and location are verified.

When the sample tracing’s non-regional portions are adjusted for normalisation by the benchmark tracing’s overall size, the samples tracing’s non-regional portions are called the false-positive region (FPR), calculated using Equation (2):

(2) FPR=100×T−O/R

The absolute variation in trace region, normalised by the original tracing total region, was the relative variance (RV), calculated using Equation (3):

(3) RV=100×T−R/R

To assess different aspects of tracing disparities, we utilised three error metrics. The ARVs evaluated the relative disparity between area measures without taking injury border location into account. However, the ARV may be modest or nil if commentators tracked totally independent regions of the same dimension. In contrast, FNR and FPR contrasted the experimental tracing and comparison tracing by assessing overlapping and underlapping, giving more information about the source of a mistake than Jaccard’s and Dice parameters, that incorporate both forms of faults. Two recordings for an image have been contrasted where only one tracing determined full skin epithelization or absence of granular tissue. However, if two or all three tracers indicated full epithelialization, no comparisons could be made for that image.

### 3.3. Identification of Tissue from Wound Imaging

Wound detection requires precise tissue separation. Complex lesions can exhibit irregular shapes, imprecise borders, and highly heterogeneous colouration [7]. To address this challenge, a convolutional network was employed to develop a system capable of classifying different tissues present in the wound. Multiple models were trained using a wound dataset consisting of 726 samples. The tissue samples in the dataset were distributed proportionally and evenly. To ensure successful outcomes, the images underwent preprocessing and augmentation steps. A black mask was applied around the ROIs; the images were resized to dimensions of 200 × 200 pixels, and they were divided into sections measuring 5 × 5 pixels. In our approach, we applied transformations such as rescaling the zoom range and performing horizontal flips during the augmentation phase. These techniques were utilized to enhance the dataset and improve the effectiveness of deep learning algorithms.

**Figure 6:**
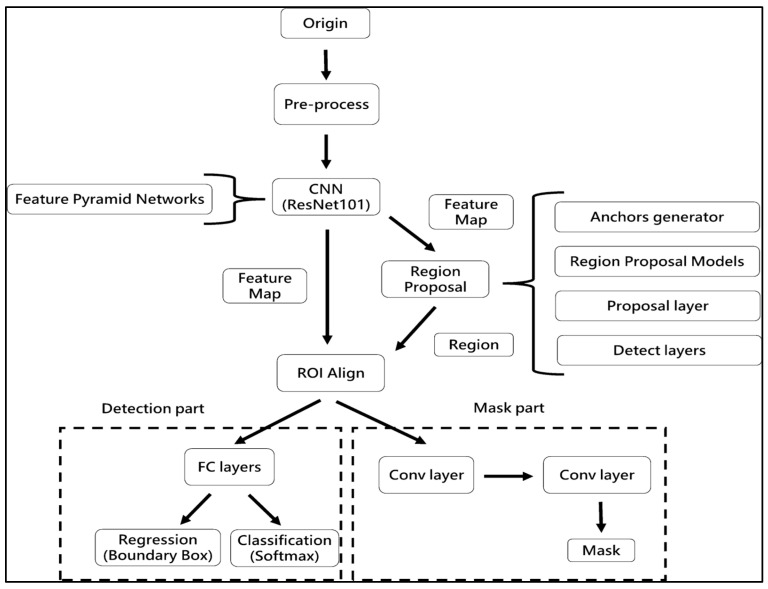
Flow chart of RA-CNN model.

The information was then divided between learning groups, which included 80% of the specimens, as well as test collections, which included the balance, or 20%, of the sample sizes. This division allowed for training the model on one set and evaluating its performance on the other. The network’s precision, memory capacity, and F1 score were analysed to assess its effectiveness. To extract features from the data, transfer learning was utilised as it is specifically designed for small training databases. Compared to models built from scratch, transfer learning offers advantages, such as requiring less data, reducing training time, and improving overall performance. This study utilised Region Based CNN, which is recognised for its outstanding image classification capability due to its many small filters that considerably decrease the total amount of parameters, though at a less rapid processing pace. It additionally introduces additional blocks through the use of intermediate layers present. To tailor this CNN algorithm to the job at hand, the information set’s picture input size was changed, and four more layers were added. These layers comprised a layer of flatten to transform the outcome of the model into a vector of attributes, a dense layer having ReLU stimulation, a layer for Dropout containing a proportion of 0.3 to avoid excessive fitting, and a heavy barrier with activation using softmax to provide a category probabilities matrix as the end product. Accurate identification of samples is a highly significant step in creating a supervised learning system [21]. Recognizing the significance of correctly categorising the dataset, a support system was introduced to assist in the labelling process. During this stage, clinical experts collaborated to assign labels to all the generated images (refer to Figure 6).

#### Region Anchored CNN

The procedure flow of the Region Anchored-CNN model framework (Figure 4) involves the following steps: ResNet101, a well-known CNNs framework in MLs, is used in the foundation to build a feature pyramidal network (FPN) to produce characteristic maps of varied sizes. The feature map from the preceding phase is used with anchoring with bounding box regression analysis to create a network of proposed regions (RPN). Utilising the results of the regression, classifier, and segmented obligations, all three sections of ROI Align—classification, regression, and segmented—are carried out. The processes of ROI alignment, loss parameter export, and image export were conducted [2]. ResNet is a commonly utilised architecture in RA-CNN for object detection and segmentation [7]. Previous research has shown that RA-CNN outperforms other deep learning architectures for wound segmentation. Extensive hyperparameter tuning was carried out to optimize the performance of the CNN model.

In this work, we did not investigate the usage of different CNNs; instead, we were primarily concerned with assessing the performance of RA-CNN for injury separation. An enhanced neural network model for categorising objects is called RA-CNN. It has the capacity to carry out examples of segmentation, regression evaluation, and categorisation all at once. As a consequence, several kinds of losses are generated throughout the period of training, making it difficult to choose the best epoch for assessing the model. RA-CNN losses fall into instruction and verification classifications, each with six subgroups. Mrcnn_class, Mrcnn_Bbox, and Mrcnn_Anch all have a direct impact on the assessment results. The best mrcnn_class efficiency was assessed in the research’s 45th period in a training of RA-CNNs representation. In the event of a tie, the period with the most contemporary period was used. Because classifications have an established baseline but extrapolation may add mistakes as a consequence of hand annotations, mrcnn_bbox and the period with the best mrcnn_anch effectiveness were chosen.

Furthermore, a method of early stopping was employed to choose participants, and epoch 33 was identified as the point to halt training. The best accuracy in categorisation and a fair degree of reliability in analysis were both shown during this specific period. Despite the fact that there may be ambiguity in calculating wound area, the choice of period is meticulously addressed to reduce mistakes when recognising sicknesses and therapeutic strategies. According to the study’s results, the appropriate assessment period may significantly affect the RA-CNN prediction accuracy and therapeutic usefulness.

### 3.4. Detecting ROI and Measuring the Area

As part of implementing and validating the area calculation algorithm using a calibrator, an expert collected 30 samples. These samples were acquired by placing a blue adhesive marker measuring 2 cm by 2 cm in the same plane as the lesion. Conventional planimetry, the Kundin technique, and digital planimetry, which makes use of photo editing software, were all used to test the preciseness of the technique. Conventional planimetry involves drawing the injury on clear acrylic. The technique’s precision was calculated by measuring the discrepancies among the tests acquired using various approaches.

Before evaluating the algorithm’s performance with real images, a laboratory study was conducted using pre-made samples. Geometric shapes were replicated on a sheet of paper using a marker to simulate these samples. Multiple captures were taken from different viewpoints to evaluate the achievable precision and error rate. An analysis among the estimated region, the region acquired using Visitrak, with data space generated through digitised planimetry, additionally employing a graphic editor, was performed in order to verify the preciseness of the readings taken. The marker’s location was further verified by contrasting its own edges and slopes.

Let us represent the wound image data as *D*, where

(4) $$D=\{(x_{i},y_{i},z_{i})\}\;for\;i=1,2,\ldots ,N$$

wherein:

*x_i_* represents the i-th wound image,
*y_i_* represents the ground truth size category label for wound *i* (e.g., small, medium, large), and
z_i_ denotes the ground truth tissue type label for wound i (e.g., granulation tissue, epithelial tissue).

The Region Anchored CNN Model can be represented by the following mathematical formulation:

- Feature Extraction: *F(x_i_)* denotes the feature extraction process, which transforms the input wound image x_i into a feature map *F(x_i_)*.
- Region Proposal Network (RPN): *R (F(x_i_))* generates region proposals (ROI) based on the featured maps *F(x_i_)*, denoted as *R (F(x_i_))*.
- ROI Alignment: The featured mapping *F(x_i_)* and ROI proposals *R(F(x_i_))* undergo ROI alignment to extract aligned features for further processing.
- Size Classification: *S(R(F(x_i_)))* represents the size classification branch, which takes the aligned features and predicts the size category label *y_i_* for wound *i*.
- Tissue Classification: *T(R(F(x_i_)))* represents the tissue classification branch, which takes the aligned features and predicts the tissue type label *z_i_* for wound *i*.
- Loss Functions: The model is trained using appropriate loss functions for size and tissue classification tasks. Let *L_s_* and *L_t_* represent the size and tissue classification loss functions, respectively.

The overall objective function for training the Region Anchored CNN Model can be expressed as follows:

(5) $$J=\Sigma [i=1\;to\;N](L_{s}(S(R(F(x_{i}))),y_{i})+L_{t}(T(R(F(x_{i}))),z_{i}))$$

where *J* represents the overall loss function to be minimized during training, and the summation is taken over all N wound images in the dataset *D*.

The equations for the size and tissue classification tasks using cross-entropy loss functions are as follows:

Let *x_i_* be the input wound image, *F_R_(x_i_)* be the aligned features obtained after ROI alignment, and *P_s_(y_i_ | F_R(x_i_))* be the predicted probability distribution over size categories for wound *i*.

The cross-entropy losing a function:

(6) $$L_{s}(y_{i},P_{s}(y_{i}|F_{R}(x_{i})))=-\Sigma [j=1\;to\;N_{s}]y_{i,j}\times log(P_{s}(y_{i,j}|F_{R}(x_{i})))$$

wherein:

*y_i_* is the ground truth one-hot encoded vector representing the size category of wound i.
*N_s_* is the no. of size categories.
*y_i,j_* is the *j*-th element of the ground reality size category vector *y_i_*.
*P_s_(y_i,j_ | F_R_(x_i_))* is the predict prospect for the *j*-th size category for wound *i*.

For tissue, Classification representation: let *P_t_(z_i_ | F_R_(x_i_))* be the predicted probability distribution over tissue types for wound i.

The cross-entropy losing a function:

(7) $$L_{t}(z_{i},P_{t}(z_{i}|F_{R}(x_{i})))=-\Sigma [k=1\;to\;N_{t}]z_{i},k*log(P_{t}(z_{i},k|F_{R}(x_{i})))$$

wherein:

z_i_ is the ground truth one-hot encoded vector representing the tissue type of wound i.
N_t_ is the no. of tissue types.
z_i_,k is the k-th element of the ground reality tissue type vector z_i_.
P_t_(z_i,k_ | F_R_(x_i_)) is the predicted probability for the k-th tissue type for wound i.

These equations represent the standard cross-entropy loss functions for multi-class classification tasks, such as size and tissue classification.

## 4. Empirical Analysis of the Proposed Methodology

The subsequent sections will present the results obtained from different stages of the research. To ensure the credibility of the findings, a comparison was made with results from previously established and validated methodologies.

### 4.1. Detection of ROI and Region Quantity

Super cells and K-mean methods were compared for rapidity, precision, and efficacy in segmentation. 

Initially, a projection analysis was conducted, utilizing a predetermined number of segments (N) and a fixed parameter (Sigma), through manual assignment. After evaluating the obtained outcomes, we recommend that polynomial regression be employed for optimal calculation in different scenarios. Specifically, using 50, 100, and 200 segments (N) and 1, 3, and 5 Sigma values, it was found that the combination of 100 Super pixels (SP) and Sigma 3 produced the most favourable results. For the inadequately segmented images, a selection was made to reprocess them using Super pixels with alternative values of 150, 200, and 300. To estimate the appropriate N segments and Sigma values, a polynomial regression approach is proposed, taking into consideration the diverse types of wounds and their resolutions. Initially, a projection analysis was carried out by manually assigning a fixed number of segments (N) and a parameter (Sigma). After analysing the obtained outcomes, polynomial regression is recommended for optimal calculation in each scenario. Specifically, using 50, 100, and 200 segments (N) and 1, 3, and 5 Sigma values, it was determined that the combination of 100 Super pixels (SP) and Sigma 3 achieved the most favourable results as in Table 2.

For the images that were not adequately segmented, a subset was chosen for reprocessing using Super pixels with alternative values of 150, 200, and 300. To estimate the appropriate number of segments (N) and Sigma values, a polynomial regression approach is proposed, taking into consideration the various types of wounds and their resolutions.

**Figure 7:**
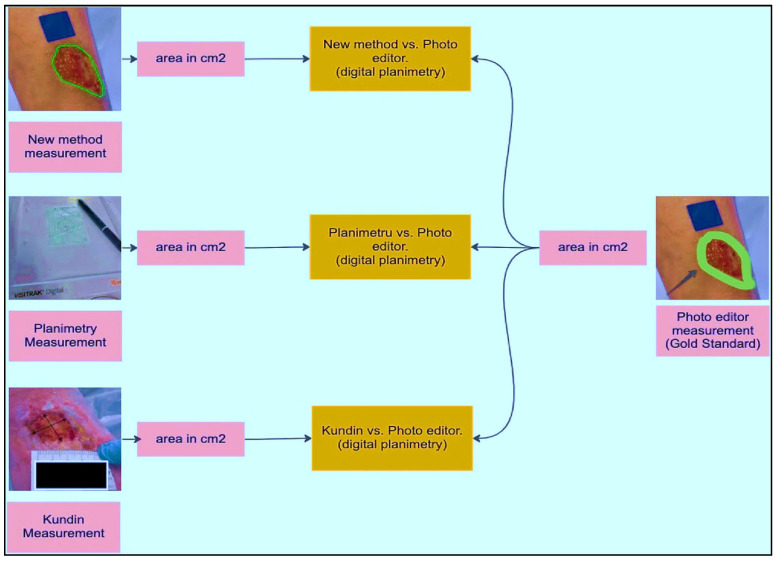
Proposed model, planimetry, Kundin, and photo editor comparison.

A desktop application was employed by a clinical expert to accurately outline the shape under high-resolution conditions. The medical expert measured the identical incisions utilising planimetry and a Visitrak instrument as in Figure 7, which were then utilised to determine the area and to verify the technique’s efficacy. Height and width were measured using the Kundin measure. When calculating the REs of the measurements, the total variance was subtracted from the result of the expert’s Digital Tool. Groups of data were established. The null hypothesis asserts that the median variations are insignificant whereas the alternative explanation argues that some are of statistical importance.

**Figure 8:**
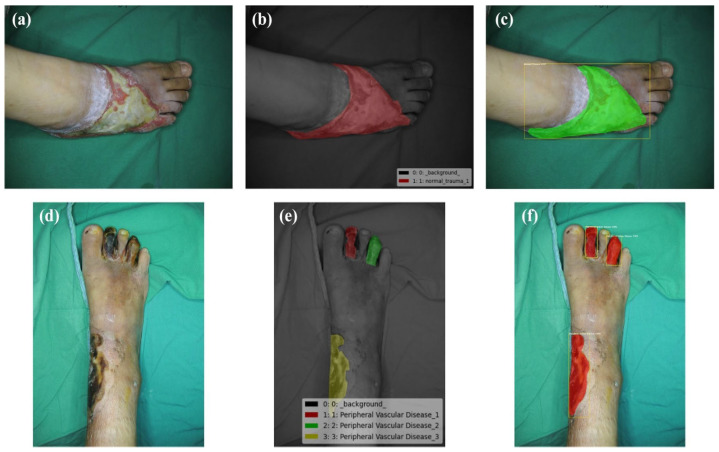
Outcome of the proposed result. (**a**) Normal wound of trauma, (**b**) Ground truth, (**c**) Superpixel contour, (**d**) RoI phase, (**e**) Tissue classification, (**f**) RA-CNN results.

**Figure 9:**
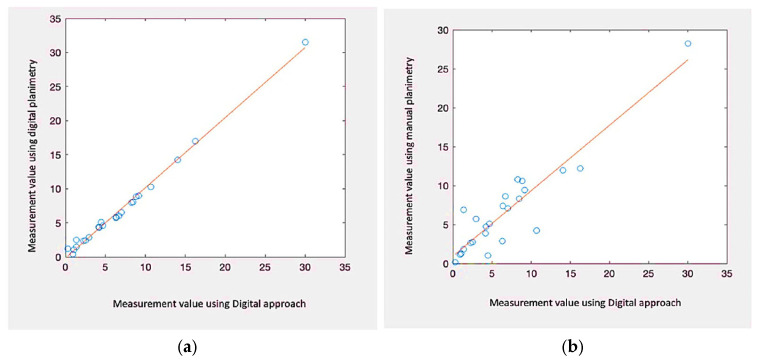
Correlating Contour vs. area result. (**a**) Proposed vs. digital planimetry of n = 28. (**b**) Proposed vs. manual planimetry of n = 28.

We can dismiss the null argument that the variances in the median of REs are not statistically important if the *p*-value is 0.05 or lower. This proves that the system utilised affects the region being measured numerically. We ran tests with p-values larger than 0.05 to determine whether there was a statistically significant disparity among the mean of various groups, that were categorised by injury length as in Figure 8. Due to the data’s non-normal dispersion and different group dimensions, we used Matlab’s Kruskal–Wallis statistic and ANOVAs. The data acquired using conventional planimetry, the Kundin technique, and our Digital approaches methodology all showed substantial variations in the findings (*p*-value 0.04 in the case in all instances). High reliability between ratings, with values ranging 0.99 to 1.00, appeared as well for every metrics. Consequently, considering digital planimetry as the most accurate reference, our proposed method demonstrates the closest relative variance, as in Table 3. For qualitative factors, absolute percentages were calculated. In this research, repeated measurements of injury pictures are symbolised by the ICC, which stands for (Intraclass Reliability Correlation). *p*-values under 0.05 were taken into account when determining statistical significance (shown in Figure 9).

### 4.2. Tissue Classification

**Figure 10:**
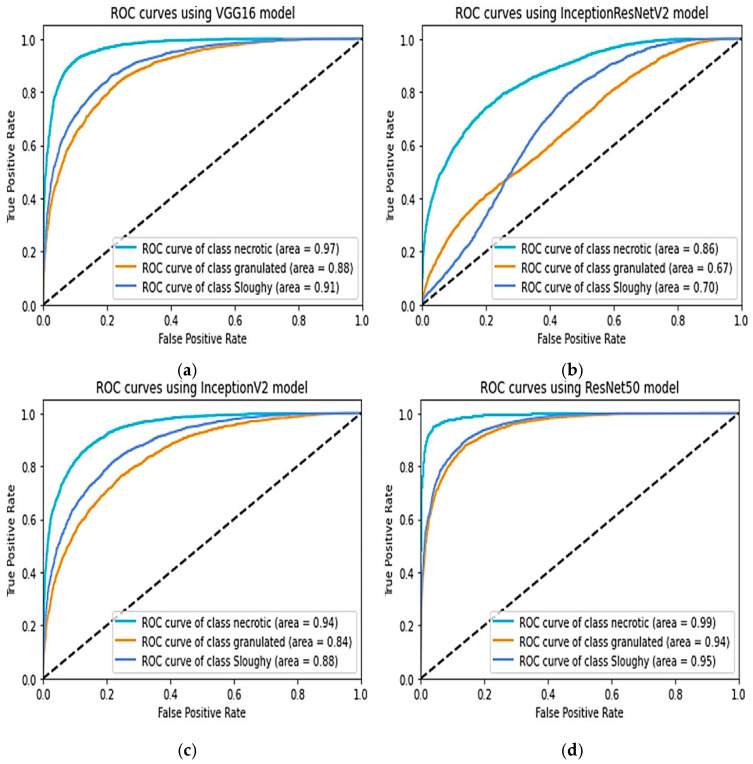
Comparison of ROCs, (**a**) VGG16, (**b**) InceptionV2, (**c**) InceptionV3, (**d**) proposed model.

Evaluating the equation used in the pre-trained boxes and our network’s parameters, the table below shows tissues categorisation accuracy. The whole collection was separated into a training collection that had 80% of the information (n = 727) with a set to be tested that contained 20% of the information after being normalised to guarantee a comparable proportion of every tissue type. The proposed model exhibited the highest performance for both training and testing, achieving an accuracy of 85% during training and 99% in predicting necrotic tissue. Except for InceptionResNetV2, that produced a success rate of 49%, each of the other models had the best achievement, with an efficiency of more than 70%. The ROC curves indicated that necrotic tissue consistently had the highest degree of accuracy in every instance, meaning it was the tissue class most accurately predicted by the models as in Figure 10.

In terms of classification performance, the enhanced RA-CNN architecture utilizing the LRELU activation function demonstrates superior overall performance when applied to the transfer learning of the small wound dataset.

### 4.3. Discussion

Various methods [7,22,23,24,25,26] have been examined to simplify and expedite the assessment of wounds by medical professionals compared to alternative approaches. A decision-support technology [27,28,29] that incorporates artificial vision with AIs features may aid in the categorisation of the injury and cautious, assisting in avoiding complicated operations even if the healthcare professional continues to have the ultimate say. In terms of accuracy, noncontact techniques for evaluating patient lacerations were found to outperform other methods, including conventional techniques. Our approach combines the identification of relevant areas, calculation of their size, and classification of the tissue involved. Supplementing the evaluation with photographs taken from different perspectives is essential. Placing the marker incorrectly can also introduce challenges in accurately measuring the wound. The doctor must take the shot at a 90-degree angle and keep the marker in the identical planes as the image, even if systems could identify aberrations. There may be major computing challenges if the marker shifts and ends up on another surface. In addition, brightness has to be considered. Tissue categorisation algorithms may make mistakes if specimens are taken under different illumination circumstances.

Despite these hazards, we think improved artificial intelligence and computational learning techniques will ultimately have an integral part in the practice of medicine. This is primarily due to the value these tools bring to healthcare professionals and the overall improvement they offer in patient care.

## 5. Conclusions

In this study, different computer vision-based algorithms were evaluated to perform area calculation, boundary determination, and tissue classification of wounds using cost-effective hardware. The results showed that a smartphone can produce reliable findings, which can be beneficial in clinical practice. The categorisation of the material around the injury and appraisal of the injured region provides medical experts with in-depth knowledge about the injury’s present condition and probable future development. These variables are crucial in defining the concept of healing time, understanding the factors influencing the healing process, and assessing treatment effectiveness. Furthermore, the RA-CNN learning model was trained and successfully produced tissue categorisation results. The digitization of tissue quantity in a wound represents a significant advancement in wound treatment. Identifying necrotic cells is particularly vital during the healing process, as it determines the need for appropriate treatment. The trained RA-CNN model achieved an RV score of 0.01–15.1 during evaluation, with overall testing results indicating high recall (0.90), precision (0.96), F1 score (0.90), and Overall Accuracy (96.7). These results show the RA-CNN model’s capability to identify and categorise diseases, resulting in an efficient and convenient tool for doctors to raise the bar for therapeutic healing of wounds.

Future work: To implement AI-assisted surgical wound closure systems that analyse wound characteristics in real-time during surgery, guiding surgeons to achieve better wound closure and reduce post-operative complications.

## Figures and Tables

**Table 1:** Overview of various research studies and their approaches/models, dataset sizes, classification tasks, and performance measures.

Research Study	Approach and Model	Dataset Size	Task	Performance Measures
Abubakar et al. [10]	Pre-trained deep learning architectures (VGG-face, ResNet101, ResNet152) combined with SVM classifier	990 sample images	Binary classification (burn vs. pressure), three-class classification (burn vs. pressure vs. healthy epidermis)	Accuracy: 0.85%, Median Deviation Error: 2.91, Precision: 0.96%
Goyal et al. [11]	Conventional machine learning (BayesNet, RFs, MLPs), deep learning (InceptionV3, ResNet50, InceptionResNetV2), ensemble CNNs	1459 DFU images	Binary classification (ischemic vs. non-ischemic ulcers, infected vs. non-infected ulcers)	Accuracy (Ensemble CNN): 90% (ischemia), 73% (infection)
Dhan et al. [12]	Stacking ensemble model based on multi-mRNA data	397 wound images	Wound age estimation	Accuracy: 92.5%
Wang et al. [13]	Overview of clinical examinations	300 images	Evaluation of arterial and venous insufficiency wounds	Accuracy: 85%, Precision: 82%, Recall: 75%
Shenoy et al. [14]	Modified VGG16 network (WoundNet), ensemble model (Deepwound)	1335 wound images	Multiclass classification (nine labels)	Accuracy range: 72% (drainage)—97% (steri strips), Wound class: 82%
Alzubaidi et al. [15]	DFU_QUTNet	1609 patches	Binary patch classification (normal skin vs. diabetic foot ulcer)	Max F1-Score: 94.5%
Rostami et al. [16]	DCNN based classifier	538 wound images	Multiclass classification (surgical, diabetic, venous ulcers)	Max accuracy: 96.4%, Avg accuracy: 94.28% (binary), Max accuracy: 91.9%, Avg accuracy: 87.7% (3-class)
Sarp et al. [17]	Explainable artificial intelligence (XIA) strategy using VGG16 network	8690 lesion images	Multiclass classification (diabetic, lymphovascular, pressure injury, surgical)	Avg F1 score: 0.76

**Table 2:** Analysis of the suggested algorithm with regard to trained algorithms.

Tissue Classification	Precision	Recall	F1-Score	Support	Precision	Recall	F1-Score	Support
	VGG16				InceptionResV2			
Necrotic	85	88	87	3026	55	85	67	3026
Slough	74	68	70	3026	43	59	50	3026
Granulated	73	77	75	3026	42	4	7	3026
Macro average	77	77	77	9078	47	49	49	9078
Overall Accuracy	77	78	78	9078	47	49	41	9078
	InceptionV3				Proposed			
Necrotic	84	77	80	3026	96	90	93	3026
Slough	67	64	65	3026	79	83	81	3026
Granulated	67	75	71	3026	82	83	82	3026
Macro average	72	72	72	9078	86	85	85	9078
Overall Accuracy	72	72	72	9078	96.7	85	85	9078

**Table 3:** Computation of errors.

Models	Mean	Median	RV	95% CL of ICC
Proposed	2906	1.80	0.01–15.1	98
Planimetry	15.7	9.3	0–75	95
Kundin	22.01	23	2.9–65.5	92

## Data Availability

The data presented in this study are available on request from the corresponding author.

## Nomenclature

FNR	False Negative Rate
FPR	False Positive Rate
RV	Relative Variance
D	Dataset containing wound images
N	Total number of wound images in dataset
*x_i_, y_i_, z_i_*	Input wound image, Ground truth size category, Ground truth tissue type for wound *i*
R	Number of relevant instances in dataset
O	Number of observed instances in dataset
T	Total number of instances in dataset
J	Overall loss function to be minimized during training
*P_s_(y_i_ | F_R_(x_i_))*	Predicted probability distribution over size categories for wound *i*
*L_s_(y_i_,P_s_(y_i_ | F_R_(x_i_)))*	Cross-entropy loss function for size classification
*y_i,j_*	*j*-th element of the ground truth size category vector *y_i_*
*N_s_*	Number of size categories
*P_t_(z_i_ | F_R_(x_i_))*	Predicted probability distribution over tissue types for wound *i*
*L_t_(z_i_,P_t_(z_i_ | F_R_(x_i_)))*	Cross-entropy loss function for tissue classification
*z_i_,k*	*k*-th element of the ground truth tissue type vector *z_i_*
*N_t_*	Number of tissue types
